# Holistic precision wellness: Paving the way for next‐generation precision medicine (ngPM) with AI, biomedical informatics, and clinical medicine

**DOI:** 10.1096/fba.2024-00198

**Published:** 2025-03-03

**Authors:** Sawsan G. A. A. Mohammed, M. Walid Qoronfleh, Ahmet Acar, Nader I. Al‐Dewik

**Affiliations:** ^1^ Qatar University QU Health, College of Medicine Doha Qatar; ^2^ Healthcare Research & Policy Division Q3 Research Institute (QRI) Ypsilanti Michigan USA; ^3^ Department of Biological Sciences Middle East Technical University Ankara Turkey; ^4^ Department of Pediatrics, Women's Wellness and Research Center Hamad Medical Corporation Doha Qatar

**Keywords:** artificial intelligence, biomedical informatics, lifestyle medicine, multi‐omics, P4 medicine, precision medicine, wellness

## Abstract

A “quiet revolution” in medicine has been taking place over the past two decades. There are two converging dynamic forces that have propelled precision medicine to the limelight, garnering wide public attention. The first driver is the realization that populations within a disease area can be stratified, thus developing therapies tailored to their specific needs, and the capability to identify these populations by analyzing large, diverse datasets. The second driver is technology advances in multi‐omics approaches and applications (i.e., molecularly informed medicine) enabling a more comprehensive portrait of disease biology. This promises to not only accelerate the development of precision medicine processes but also presents challenges for healthcare professionals and health systems that are struggling to interconnect and integrate disparate data sources into a cohesive clinical strategy to the benefit of their patients. We coin here the term next‐generation precision medicine (ngPM), which is bound to become conventional in the clinics sooner or later. Artificial intelligence (AI) and machine learning (ML) in healthcare have transformative potential and are a strategic response to today's challenges and tomorrow's opportunities. The chief challenges here are how well precision medicine (PM) permeates primary care to become a standard of care and drive toward precision wellness or precision lifestyle (ngPM), while ensuring access to care is feasible, streamlined, and routine. We present here a perspective that would harness the power of ngPM for precision wellness.

## Graphical abstract

The future of medicine—P4 medicine. Medicine that is patient‐centered, data‐driven care, which is predictive, preventive, personalized, and participatory (P4 medicine) toward accurate lifestyle and precision wellness. Precision wellness is facilitated/powered by AI; thus, it is defined quantitatively. Input parameters are wide‐ranging encompassing not only the human body (such as anthropometric, clinical & biochemical, multi‐omics, nutrition/diet, etc. measurements) but also the mind and spirit of the person.

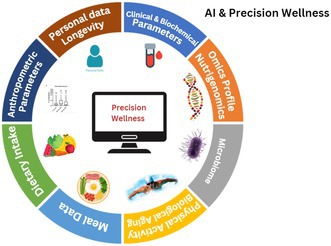

## 1 INTRODUCTION

Precision medicine (PM) and health may seem a contemporary concepts—or at least that is how they are often portrayed.^1^ However, there is an ancient precedent. Two famous quotes attributed to the “Father of Medicine” Hippocrates (400 BC),^2^ and the physician Sir William Osler (1892),^3^ considered the “Father of Modern Medicine” and one of the founding professors of Johns Hopkins Hospital, reflect this notion. The former had stated “It's far more important to know what person the disease has than what disease the person has” while the latter remarked “If it were not for the great variability among individuals, medicine might as well be a science, not an art”.

The convergence of multisystem medicine and technological advancements in molecular medicine has paved the way for what we term “next‐generation precision medicine” (ngPM)—a Quality‐of‐Life (QoL)‐driven approach. This evolution emphasizes patient‐centric treatment that is equitable and timely for those who are ill, while shifting focus toward precision wellness management for healthy individuals, making wellbeing the central objective. By tailoring care to each individual's unique needs, ngPM strives to enhance both treatment outcomes and overall QoL. In essence, ngPM strives to improve precision health and personalized care. It is an integrative personal profile and a holistic QoL approach. This deep health state results from the interplay of six elements: physical, emotional, mental, social, environmental, and existential/spiritual. Artificial intelligence (AI) aids this process by analyzing “omics” and deep phenotypic data, encompassing not only the human body but also the mind and spirit of the person.^4^

While PM offers considerable opportunities for healthcare, these opportunities also present challenges. The dawn of individualized medicine was further enabled by substantial developments in the field of health information technology (HIT), including information management systems (IMS).^5^, ^6^ A significant achievement in this area worthy of mention entails the electronic storage and processing of patients' data, facilitating the clinical adoption of the PM concept, particularly through translational and clinical research. The implementation of electronic health records (EHRs)—which archives and stores information on patient history, medications, test results, follow‐ups, and demographics—has been crucial in integrating datasets derived from genetics and genomics research within clinical settings.^5^, ^6^, ^7^

In view of the accelerated pace of healthcare discoveries, big multidimensional data generation streams—whether quantitative or qualitative in nature—are expanding along three vectors [variety, volume, and velocity] and presenting exceedingly complicated relationships^8^; hence, making AI an imperative due to its processing power and speed.^5^, ^6^, ^9^ This vast trove of available data one cannot untangle the complexity to make sense of it without mining it using appropriate analytical tools and computational analyses. AI predictive power enhances routine medical decisions, diagnostics, laboratory results interpretation, etc. among other applications.^9^

**FIGURE 1:**
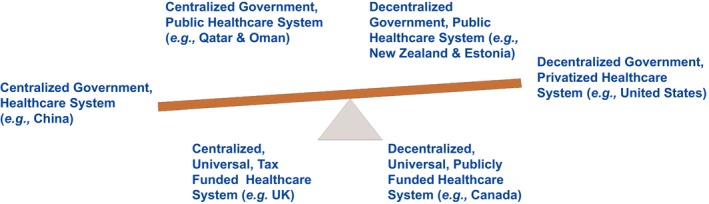
Healthcare continuum. Precision healthcare and precision public health elevate healthcare systems at the population level, with a focus on tailoring interventions. This continuum, based on the Information Management Systems (IMS) framework,^6^ illustrates how healthcare models range from centralized to decentralized structures per geographical region. The “balancing saw” graphic emphasizes the delicate equilibrium between government involvement, funding mechanisms, and cultural/ethnic influences that shape each healthcare system.

Drawing on over 80 years of collective healthcare experience across diverse systems in North America, Europe, and the Middle East, we offer our reflections on the value of the work done in this field. Our insights also consider the cultural and ethnic predispositions of the populations we have served. Figure 1 captures this perspective, illustrating how healthcare systems range from centralized to decentralized, with funding and insurance preferences shaped by cultural factors [The Economist Intelligence‐EIU].^8^

We present here a conceptual framework for ngPM. The opportunities or benefits for ngPM arise through the union of AI, biomedical informatics, and clinical medicine. These benefits can be grouped into three categories elaborated on below.

(1) Benefits at the patient level—PM enables earlier disease diagnosis and patient management. It also provides therapeutic options, risk assessment, and drug safety profiling, leading to better clinical decisions and improved patient outcomes^10^, ^11^ throughout the lifespan. For example, PM has revolutionized drug discovery and the pharma industry,^12^, ^13^ facilitated genomic newborn screening for diseases,^14^ and advanced longevity research (healthspan/lifespan).^15^ While PM leans more toward therapeutic interventions, precision wellness (ngPM) emphasizes scientifically guided lifestyle intervention. It is here where AI bridges the information gap to afford better person health care.^16^

(2) Benefits at the population level (especially, in the different geographical regions, countries)—PM has been a key driver in shaping national healthcare strategies and policies, significantly impacting public health.^17^, ^18^ The public health sector presents immense opportunity for precision healthcare (patients stratification and informed clinical decisions, i.e., the right treatment for the right person at the right time in the right place) and precision public health (the application of new technologies to public health policy and practice, i.e., the right intervention to the right population at the right time) to elevate the healthcare system service at a population level (Figure 1), which illustrates the importance of IMS, that is, information on government‐based management systems and their health care policies. Despite the massive amount of health and medical data generated daily, much of it remains underutilized. AI‐driven big data analysis enables the identification of patterns, variant filtering, drug target discovery, phenotype–genotype correlations, gene‐disease associations, and advancements in pharmacogenomics, among other applications. The COVID‐19 pandemic exemplifies the power of this approach.^19^ From tracking the virus's spread to analyzing population responses, it catalyzed unprecedented collaboration between scientific research and public health communities. Ultimately, it opened new avenues for pathogen identification and monitoring, demonstrating the immense potential of AI in managing global health crises.

A valuable tool and enabler in this domain is digital twin,^20^ which is a virtual model mirroring a physical entity where one can simulate an individual's unique physiology and biochemistry, multimodal interventions, etc., thus driving a spectrum of transformation from drug discovery and development to healthcare management to improve population health.

**FIGURE 2:**
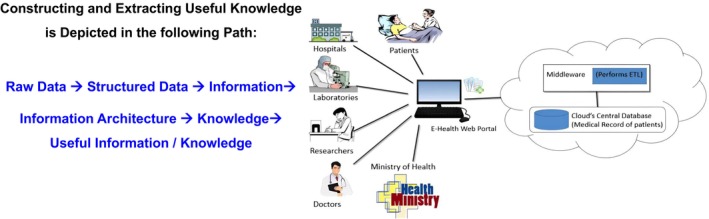
Information management path. Converting datasets into useful knowledge to improve healthcare. Adapted from Sarwar et al. 2019 and Zhai et al. 2022.^6^, ^21^ Data science is instrumental in transforming raw data into quantifiable data leading to insights (useful information/knowledge) which are delineated in the left‐side pathway. Scalable infrastructure, fast connectivity, and cloud computing, shown in the right‐side panel, are critical and integral pieces for real‐time large data exchange among participants like hospitals, clinical laboratories, patients EHR, public health entities, etc. that possess specific information architecture (patient record database) per their function that is accessed via a portal.

(3) Benefits at the health system level—PM has been a driver for innovation and helping manage data of the healthcare system.^6^ There is tremendous value in harnessing such data to benefit both patients and populations (Figure 2). The key to tackling healthcare's biggest challenges is the ability to transform the vast amounts of data into rich and meaningful insights.^5^, ^6^ This knowledge pathway is shown in Figure 2. By leveraging AI and machine learning (ML) tools, we can create comprehensive Healthcare Maps that transform raw data into actionable insights, facilitating knowledge translation. These maps provide a precise view of the healthcare system by enabling advanced disease surveillance and signal detection, predicting and managing risks, targeting treatment interventions for specific subpopulations, and deepening our understanding of various diseases.^6^ In this regard, evidence‐based decision‐making at the healthcare system level results in more accurate interventions and more effective approaches to disease diagnosis, monitoring, and treatment.^8^, ^22^ Ultimately, this enhances the quality of treatment, making healthcare more value‐based and economically sustainable.^23^

A notable example of data transformation at both the population and healthcare system levels (transformation of data→ information→ applicable/implementable useful knowledge) is the Blue Cross Blue Shield (BCBS) medical map. BCBS, a US‐based health insurance provider, offers a nationwide, personalized approach to healthcare. One of its programs, the Health of America Initiative, uncovers and shares key healthcare trends through advanced analytics and innovative research. The BCBS Health Index^SM^ quantifies the impact of over 200 medical conditions on the health and well‐being of commercially insured Americans. Its recent data show that hypertension, major depression, high cholesterol, coronary artery disease, and type 2 diabetes are the top five health conditions that adversely impact Americans' quality of life and longevity.^5^, ^6^, ^24^

Looking at PM, the strongest impact made has been in the area of oncology^10^, ^11^; also it made an obvious difference in the diagnosis of genetic rare diseases, pharmacogenomics, and in the areas of pathogen identification and monitoring [The Economist Intelligence‐EIU].^8^ The latest advances involve using AI to expedite genome interpretation and nominate candidate diagnoses for rare genetic and/or newborn diseases. The cost of interpreting genomic reports has now become the largest single expense. The process of understanding genetic variants is highly manual, and the volume of data can be overwhelming. Fabric GEM developed by Fabric Genomics [Fabric Genomics] and other like tools such as Moon developed by Diploid Genetics [acquired by Invitae and later by Labcorp] offers a fully automated solution, delivering results quickly. It assists clinical geneticists in diagnosing by using AI to analyze data from clinical notes, medical databases, and genome sequences specific to a patient. This AI tool leverages its training on similar data to identify connections with over 6700 known disease‐associated genes.^25^

GEM integrates two primary AI algorithms that are already in use by human interpreters: Phevor (Phenotype Driven Variant Ontological Re‐ranking Tool), which identifies and ranks the clinical features of genetic diseases based on the observed phenotypic characteristics in the patient [Yandell Lab], and VAAST (Variant Annotation, Analysis & Search Tool) optimizer/prioritizer [Yandell Lab], which ranks genetic variants according to their likelihood of causing disease, from “benign” to “definitely disease‐causing.” Additionally, Natural Language Processing (NLP) can be added to GEM to extract critical clinical features from EHRs, removing another manual step in the process.^25^

Clinical genomics or next‐generation sequencing is key to advancing ngPM and is gradually being adopted by hospitals. AI‐driven decision support tools accelerate the interpretation of newly available genomic data by evaluating large structural variants, variant quality, deleteriousness, prior clinical annotations, and modes of inheritance. Gene scores further summarize the strength of evidence indicating whether a gene is damaged and how this damage might explain the observed phenotype.

Below, we discuss two case studies that highlight progress in using AI‐based clinical decision support tools. The rationale for selecting these case studies is twofold: (1) There is an unmet need for patients in this area, particularly in developing countries, and (2) ngPM can have the most significant impact when implemented in these settings.

### 1.1 Case study 1—pediatric cancers

Pediatric cancers have an overall low incidence rate. However, in the USA among children aged 0–19 years, pediatric cancers are the leading cause of death.^26^ For a decade now, ca. 300 K children are diagnosed with cancer globally every year.^27^, ^28^ Amid children aged 0–14 years, the predominantly diagnosed childhood cancers were leukemias, lymphomas, neuroblastomas, and brain/CNS tumors, unlike the adolescent age group.^27^ In the USA, the death rate for the combined children and adolescent age group was approximately 25 per million.^29^, ^30^ Data analysis for the period 2001–2016 indicates that the highest mortality rate noticed is associated with leukemias (28.5%), brain and CNS (26.9%), and bones and joints tumors (9%).^29^, ^30^ In the period 2016–2022, a decrease of ~1.5% was observed in the mortality rate that largely was attributed to the accessibility of advanced treatments and supportive care for pediatric leukemia and lymphoma.^31^ On the other hand, soft‐tissue, brain, and bone cancers mortality rate remained unchanged.

Pediatric Cancer Predisposition Syndromes (CPSs) are defined as “a group of genetic disorders characterized by an augmented risk of developing pediatric cancers”. CPSs are typically induced by inherited genetic mutations in specific cellular pathways genes. Several of these genes have been linked to DNA repair mechanisms, cell cycle regulation, or tumor suppression biology. Some representative cases include Li‐Fraumeni syndrome, neurofibromatosis type 1, hereditary retinoblastoma, familial neuroblastoma, and familial adenomatous polyposis, among others.^14^, ^32^ Yet, clinical genomic studies judge that roughly 10% of pediatric cancer patients have an underlying cancer predisposition syndrome. Moreover, a high percentage of pediatric cancers are accompanied with germline mutation in CPS genes.^33^, ^34^, ^35^

Early detection of cancer offers numerous benefits to patients, like effective treatment that leads to better survival and, all in all, less harm or misery.^36^ Genomic sequencing of newborns has been suggested to be an appropriate method for first screening and, in the long run, foreseen to become an integral part of any newborn screening program.^37^, ^38^, ^39^ The next‐generation sequencing (NGS) tool has been applied in quite a few newborn screening programs for years now. For instance, using targeted gene panels, whole exome sequencing (WES), or whole genome sequencing (WGS) approaches.^40^, ^41^, ^42^, ^43^, ^44^, ^45^, ^46^ A main advantage of newborn screening targeted gene panels is the ability to be customized [what is called “on‐demand” design] to augment the genomic library with specific/targeted gene regions to be sequenced.^47^, ^48^ Attaining quality read depth and high coverage of large to medium‐sized gene panels allows the identification of significant single nucleotide variants (SNVs), small indels, and copy number variants (CNVs) overlapping the gene panel of interest.^46^ Incorporating genomics into large‐scale newborn screening programs^37^, ^38^ could significantly improve the early detection of treatable rare diseases and CPS.^49^, ^50^, ^51^, ^52^ Besides, the availability of this genomic data can deliver strategic health benefits, help forthcoming research activities, and formulate healthcare policies.

Finally, in this AI era, there is a tremendous advantage that can be gained when it is applied to clinical cytogenetics. Some of the benefits include: improved diagnosis, robust analysis, enhanced digital workflow, facilitate better standardization and quality control processes, and build system efficiencies (mitigate errors and enhance overall outcomes).^53^, ^54^

### 1.2 Case study 2—rare diseases

Rare diseases affect 30 million people in the USA and more than 300–400 million worldwide.^49^ Rare inherited diseases exemplify the healthcare burden both globally and in the Gulf Cooperation Council (GCC) region specifically due to the high consanguinity rate.^55^, ^56^, ^57^ By some estimates, about 8% of infants in the Gulf region are born with some type of a genetic disorder.^55^, ^56^, ^57^, ^58^ Deploying NGS in the clinics (clinical genomics) has ushered in a new era of genomic medicine to support patient diagnosis and care^37^, ^38^, ^59^, ^60^ including the GCC.^61^, ^62^ Ultra‐(rapid) genome sequencing is a paradigm shift both in terms of extremely rare cases or undiagnosed diseases from faster treatment, right up to population‐level systemic changes^63^ to national and international healthcare policy.^17^, ^64^ AI approaches are central to this advancement to automate and accelerate genomic analysis where the workflow is optimized for speed, accuracy, and reporting. The timely delivery of tailored results informs medical management, expedites disease‐specific molecular diagnoses, and equips clinicians with insights on evidence‐based intervention options. Additionally, long‐read DNA sequencing is emerging as an important technique for rare/complex human genetic diseases to identify pathogenic mutations or structural variants, which would be applied to molecular diagnosis and therapeutic strategies for patients with genetic diseases in the future.^63^, ^65^, ^66^ As a matter of fact, rapid whole‐genome sequencing as a service for newborns with rare diseases is becoming a reality both in the UAE and Qatar.^14^, ^55^, ^62^

For other diseases like Alzheimer's Disease, currently available treatments are 70% ineffective, arthritis is 50% ineffective, diabetes is 43%, asthma 38% and anti‐depressants are 38% ineffective.^10^ If one understands the profile of diseases, and is able to “decode the biology” and how a tailored treatment can be effective or ineffective and the associated risks (such as side effects) then one can now really view the impact of PM and appreciate its value and influence in a variety of disease and therapeutic areas, both in terms of the patient and the healthcare system.^8^

PM is data intensive, especially when you start layering several of the omics platform technologies' data about a patient or a population,^67^ whether it is genomics or proteomics or metabolomics, or epigenomics. You can see the growth of the data in three vectors—volume, variety, and velocity, that is, the volume of the data, the nature and the relationships between the data, and the storage capacity needed to sort this kind of data to utilize it efficiently and effectively, such as in facilitating electronic health record data.^8^

There is an inherent power in data, and there is a need to keep it right, which is a challenge. AI provides better diagnostic capability and significantly aids in the areas of drug discovery and drug development. It is influencing medical devices, and we all know applications (apps), which are recognized as therapy tools for various diseases, recognized by the FDA in the USA.^68^, ^69^, ^70^, ^71^

## 2 BREATHING LIFE INTO DATA

The future is AI. Driven by advances in AI/ML and other big data analytics tools, healthcare is becoming more digital.^72^ Moreover, AI data analytics will inevitably help us decode biology to understand diseases better and tailor better treatment and care.^8^ The powerful combination of AI and PM undeniably are a transformative force in healthcare.^9^ The goal for healthcare management is the delivery of effective person‐centered care in an equitable and timely manner. However, fulfilling this promise will not be straightforward. Innovations within the healthcare system are challenging at the best of times, which means developing the right IMS framework is paramount.^5^, ^6^

**FIGURE 3:**
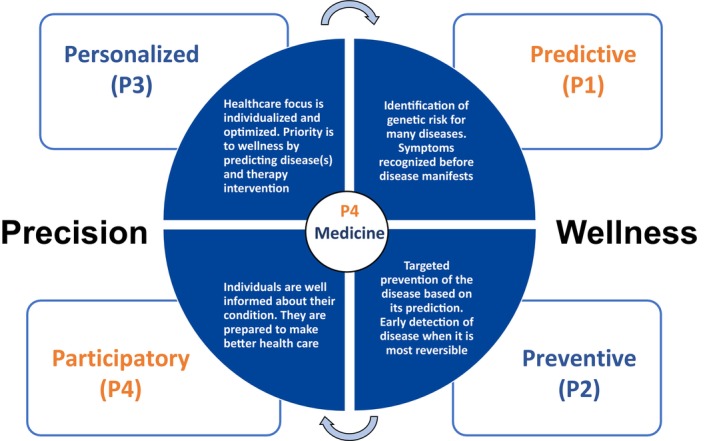
The future of medicine—P4 medicine. Medicine that is patient‐centered, data‐driven care, which is predictive (P1), preventive (P2), personalized (P3), and participatory (P4), referred to as P4‐medicine, to march toward precision wellness and QoL lifestyle. P4‐medicine is about applying ngPM “healthcare” approach to achieve precision wellness. The convergence of systems medicine (P1‐4) has circular connectivity that is also influenced by social networks and digital revolution.^73^, ^74^

Enormous amounts of health and medical records data are generated daily, with tremendous potential applications. On average, it is estimated that about 80 megabytes of information are generated per patient per year. Certainly, we can strive to optimize a person's precision health profile. While the data are there, it remains underutilized, with limited valuable information being extracted. One of the essential enablers to achieve this IMS goal is an integrated and comprehensive pathway for healthcare data storage, analysis, and utilization. Additionally, substantial collaboration between the various stakeholders in healthcare is critical to ensure the complete rollout of person‐centric care and P4 medicine. Namely, medicine which is evidence‐based care that is predictive (P1), preventive (P2), personalized (P3), and participatory (P4) (Figure 3).^73^

This notion of P4 healthcare is that it has two major thrusts—wellness and disease; the disease approach is the current traditional practice ‘sickcare’, whereas ‘healthcare’ wellness is almost entirely disregarded by the conventional medicine of the 20th century. When wellness is defined quantitatively, personal, dense, dynamic data are essential, where in‐depth analysis^74^ leads to actionable possibilities that could improve wellbeing and circumvent disease. P4 healthcare is the very essence of 21st‐Century Medicine.^73^ Therefore, the transition phase from wellness/precision lifestyle to disease and vice versa can be managed by physicians and/or patients, where intervention leads to changing this trajectory.

In our articulation of ngPM, “Precision Wellness” goes beyond P4 healthcare by becoming holistic and multidimensional by synthesizing qualitative and quantitative data sciences and by incorporating aspects such as nuances of culture and faith, interconnectedness and relationality, mindfulness and spirituality, in addition to the hard sciences associated with “scientific wellness”. The Harvard study of more than 8‐decades on happiness noted the strong role of social relationships in happiness and good health.^16^

We are fully aware of the concerns raised in implementing this approach with many being addressed along the healthcare continuum. These include “cost, data privacy, lack of racial inclusion, government control of surveillance technologies, corporate intrusion into personal health data, genetic discrimination and complexities of analyzing big data” to name a few.^73^, ^74^ We perceive a major challenge as well realizing this at the primary care level not only in terms of full incorporation but also in terms of the time that would be required for primary care physicians to apply this tactic during a patient's preventive health care visits. Furthermore, the need to manage population healthcare diversity and equity issues.

## 3 POPULATION HEALTHCARE PROJECTS

Population health endeavors in many countries were initiated by an appropriate, sizable investment in infrastructure, launching national genome sequencing projects, and establishing biobanks that are facilitated by bioinformatics, computational methods, and AI tools. Invariably, the primary focus of these efforts has been genomic medicine, that is, delivering actionable routine clinical care in rare genetic disorders, pre‐natal/newborn services, and pharmacogenomics (drug response).^75^, ^76^

**FIGURE 4:**
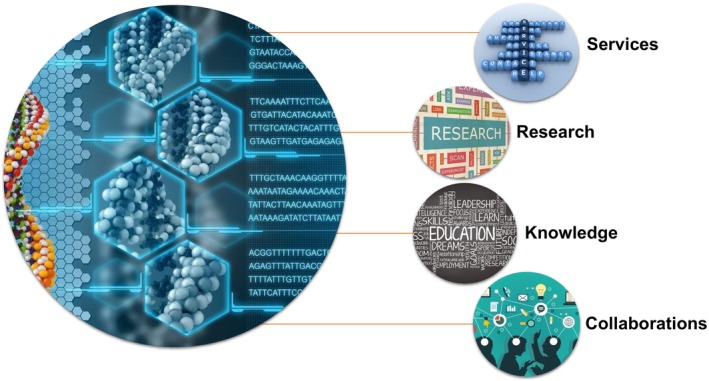
Pillars of quality of life (QoL) ecosystem. Healthcare systems, biomedical researchers, and education organizations must join forces to explore innovative precision wellness solutions. PM healthcare services offered to clinical data of individuals, biomedical research outcomes, knowledge and education, and collaborations and partnerships would harness the power of technology and innovation like high‐performance computing, AI, ML, and ngPM to drive patient outcomes by improving QoL and enhancing the delivery of public health services. The realization of this effort is supported through network ecosystem connectivity.^5^, ^6^, ^17^, ^24^

At a population level, most initial investigations have focused on either North American or Western European populations. Recognizing the limited transferability of genomic medicine, other projects were launched^77^ such as isolated populations like Iceland, Sardinia, and Faroe Islands, or small nations like Estonia and, more recently in other small countries in the Middle East and North Africa (MENA) like Qatar, UAE, and Saudi Arabia.^17^ There are population projects as well in China, Australia, and Turkey, among others. The UK leads the way with their 100,000 genomes. Today, over 85K genomes have been sequenced, mostly from NHS patients (The National Health Service‐NHS), which is the publicly funded healthcare system in England and one of the four NHS systems in the UK affected by rare disease or cancer, leading to groundbreaking insights and findings (Genomics England ^78^). In the USA, by way of example, The US Precision Medicine Initiative (All of Us Research Program—NIH trusted)^79^ aims to enroll 1 million Americans in total, who will provide personal health, socioeconomic, and sociocultural data that are complemented by biological samples and measurements, with all being linked to their EHR to create a national database that facilitates large‐scale longitudinal studies. This consortium endeavor is led by the Scripps Research Translational Institute. Last year, it has received a 5 years total funding of $282 million. Certainly, the massive integration of personalized health information and large‐scale epidemiological and molecular data (nearly 460,000 participants nationwide to date), combined with the use of AI and ML, is underway to achieve culturally and demographically relevant outcomes in PM and population health (eventually precision wellness), in ways that are practical and appeal to stakeholders. This underscores the monumental integration that is needed and should take place at the primary care level and the enigma of profit versus QoL.^80^ The purpose of this convergence is the quintessential goal of ngPM (lifestyle medicine) patient centricity in an equitable and timely manner for those who are ill and precision wellness in the case of healthy individuals (Figure 4).

Ubiquitous health (uHealth) intent is to combine extraordinary patient care, breakthrough research, and inventive education to create an innovative front‐line approach to health care (Figure 4). Health care is a continuum, and this extends to healthy individuals as well. The deep health framework is a state of being to be achieved and actually is a natural outcome of the amalgamation of population data. There are six interplay elements: physical, emotional, mental, social, environmental, and existential. The physical domain, in terms of lifelong wellness and personal wellness, is briefly summed up as lifelong wellness/lifestyle encompassing body, mind, and spirit.

It is noteworthy to mention that in parallel downstream matters need to be actively addressed for full implementation of PM and to experience its benefits at the patient level.^81^ Among these are the following: building state‐of‐the‐art technologies, developing regulations and compliance policies, structuring/expanding insurance and reimbursement coverage, protecting innovations and patents, developing human resources, ensuring sustainable levels of funding, etc. to name a few. Another set of problems to be addressed is data privacy/security concerns and ethical‐related issues, particularly in relation to genetic data. Genetic information is deeply personal and sensitive, and its misuse or unauthorized access could lead to discrimination or harm. Ensuring data privacy and security is essential to maintain patient trust and compliance with regulatory requirements. Moreover, there is a need for robust ethical guidelines to ensure that AI‐driven healthcare solutions do not exacerbate existing health inequities or biases. For further discussions, the reader is referred to these papers,^9^, ^17^, ^82^ and the EIU report [The Economist Intelligence‐EIU].^8^

Representation of human populations from various geographies and ethnicities is necessary to achieve the promises of next generation PM. An example, the H3Africa initiative “African genome project” (H3Africa)^83^ even the EU's The “1+ Million Genomes” (1 + MG) falls in this category (1+MG).^84^ Despite several new initiatives, there is still a need to cover a large and ethnically rich region like the Middle East/MENA region. To our knowledge, there is no comprehensive information on the genomes of Arab populations available at the moment (Pan Arab Genome Draft‐the UAE publication is under review DOI: 10.21203/rs.3.rs‐3490341/v1 and The Qatar Genome Project‐QGP‐ efforts Pan‐Arab‐Array).^85^ It is estimated that healthcare expenditure in the GCC reached US$ 104.6 billion in 2022. Historically, government health authorities in the GCC have functioned as regulators, operators, and insurer. National long‐term healthcare strategies are being formulated to migrate toward precision health/lifestyle medicine, to expand the role of the private healthcare sector and even becoming a medical tourism destination. The UAE is by far one of the fastest growing hubs for medical tourism globally.

ngPM and precision health globally and selectively in the MENA region since it is resources intensive as indicated above will be driven largely by AI, ML, and Quantum computing.^86^, ^87^, ^88^ Other complementary instruments include the following:

1. Automation of medical/healthcare centers
2. Digitization of electronic medical/healthcare records (EMR/EHR)
3. mHealth—mobile healthcare applications
4. uHealth (remote patient monitoring in real time using sensor technology)
5. Telehealth (telemedicine)
6. Omics‐specific signatures
7. Pre‐clinical scalable models

The chief challenges here are how well PM and lifestyle medicine permeate primary care to become a standard of care^17^, ^23^ and to what extent access to care is feasible, streamlined, and routine.

## 4 TRENDS, CHALLENGES, AND OPPORTUNITIES

Demonstrating the value of AI and ngPM is critical to garner support from healthcare decision‐makers, in particular, funders.^89^, ^90^, ^91^, ^92^ Since it requires investment in infrastructure and information management capabilities. Equally important, developing workforce and capacity‐building (training, professional development, etc.) assets for eventual and continued implementation.^92^, ^93^ In this regard, there may be a need to reform medical education and institutions to accelerate adoption.^8^ A true measure of ngPM success is the ability to take advantage of omics layers, multidimensional data integration, and assimilate into primary care services to serve patients better. In prior publications, we discussed and demonstrated how this has been applied and implemented in cancer in Qatar,^10^, ^17^, ^18^, ^94^ in the near future, data quality, standardization, and interoperability remain key to the advancement and implementation of ngPM. One aspect to this date is substantially ignored, is the consideration for culture and faith (outside diversity/equity and bioethics) incorporation into precision medicine planning and decision‐making.^17^, ^23^

AI and other complementing PM tools or multi‐omics will continue powering R&D data for the foreseeable future.^95^, ^96^, ^97^, ^98^ Additionally, it will speed up drug discovery and development.^99^, ^100^ These efforts will enhance the probability of finding novel drugs or first‐in‐class medicines. Moreover, diagnosis, predicting illness, and early warning systems will be enhanced at the population level, and there will be an inclination toward disease prevention over disease treatment; accordingly, a track en route for lifestyle medicine (Wellness). Further, it will extend to digital twins of human beings or organs,^11^, ^101^ which is a virtual model of a physical entity that dynamically pairs the physical and digital domains, hence driving the transformation of EHR and medical records and delivering highly personalized treatments, interventions, and clinical trial matching, refining patient management/care. Yet another beneficiary is reimagining pharma/biotech production and manufacturing in terms of scale, quality, and safety.

### 4.1 ngPM IN PRACTICE

The discussion here highlights the need for a paradigm shift in healthcare leaders and professionals to translate the vision into reality. A key activity is an umbrella for healthcare services, research, knowledge expansion, and extensive collaborations. It remains necessary to formulate an overarching IMS framework to unify stakeholders and collect, manage, secure, share, and maximize benefits from different health‐fused data components. Therefore, the capability to transform extraordinary amounts of data into meaningful and rich insights (information) (Figures 2 and 4). Developing such a healthcare map permits the most precise view of our healthcare system. For instance, performing disease surveillance and signal detection, risk prediction or risk management, targeting treatment interventions, understanding disease, etc.^75^, ^94^, ^102^

Implementation outlook will vary by jurisdiction because healthcare is a continuum.^23^, ^81^ The spectrum of the healthcare system ranges from fully centralized (nations with public/government health systems) to decentralized systems (nations with private health systems) (Figure 1). In any case, many questions must be answered concerning investment in infrastructure and IMS, such as who will pay for the implementation? Who will oversee the fused data macrocosm? How will patients and private entities be encouraged to buy into healthcare fusion? Among other important issues privacy and cybersecurity.

Undoubtedly, we are experiencing the “4th Industrial Revolution” per The World Economic Forum assessment. In this information age that will lead to next generation Precision Medicine & Precision Public Health, a health information center (HIC) or a national centralized database of sort, which is data‐driven, technology‐enabled and business induced is vital to make the mission a reality. Of course, this has implications at the patient and population levels. Deploying and harnessing quantum computing capability will redefine and revolutionize healthcare to solve complex problems and perform simulation. The collaboration of the Cleveland Clinic with IBM falls in this vein.^103^

At the drug discovery and development level, few things will be witnessed with respect to many improvements in biosampling, biological models, pre‐/clinical predictive models, translational potential (bench to bedside and vice versa), and clinical trials design.^93^ Ultimately, the beneficiaries are the patients with regard to highly personalized, precise therapy and overall better care, better value, better health, and better social outcomes at the healthcare system level.

## 5 CONCLUSION

While we are rapidly advancing in the field of PM, there are challenges to consider. Firstly, the importance of effective regulation and ethical considerations in PM continues to be a challenge. There is a lag in policy development and patient‐centricity. Patient information is highly personal and sensitive, and any misuse or unauthorized access can result in discrimination or harm. Protecting data privacy and security is crucial for maintaining patient trust and adhering to regulatory standards. As ngPM requires a strong infrastructure with its data and AI requirements, many healthcare professionals argue about the value. Secondly, PM has effectively led to patient engagement and patient empowerment with tailored treatment and lifestyle; however, the healthcare system still lacks expertise; the continued education of healthcare providers, professionals, and workforce is critical toward ngPM evolution. ngPM has many stakeholders that go beyond the patient and the healthcare system. There must be a stronger level of engagement and commitment in order to make PM accessible and at par with developed nations.

## AUTHOR CONTRIBUTIONS

S.G.A.A.M. performed research and collected information, and generated short write‐ups. N.I.A.‐D. and A.A. provided research insight, content examination, and supported numerous aspects during the manuscript development process. MWQ contributed to conceptual work, framework, final draft write‐up, critical reading, and editing. All authors read and approved the final manuscript.

## FUNDING INFORMATION

This research received no specific grant from any funding agency in the public, commercial, or not‐for‐profit sectors.

## CONFLICT OF INTEREST STATEMENT

The authors declare that there are no conflicts of interest or competing interests.

## Data Availability

This is an opinion, literature‐based review article. No new data were created or analyzed in this study. Data sharing is not applicable to this article.
